# Supplementary figures and images for: Dementia and patient outcomes after hip surgery in older patients: A retrospective observational study using nationwide administrative data in Japan
Source: PLoS One. 2021 Apr 22;16(4):e0249364. doi: 10.1371/journal.pone.0249364 (PMC8061936; doi:10.1371/journal.pone.0249364)

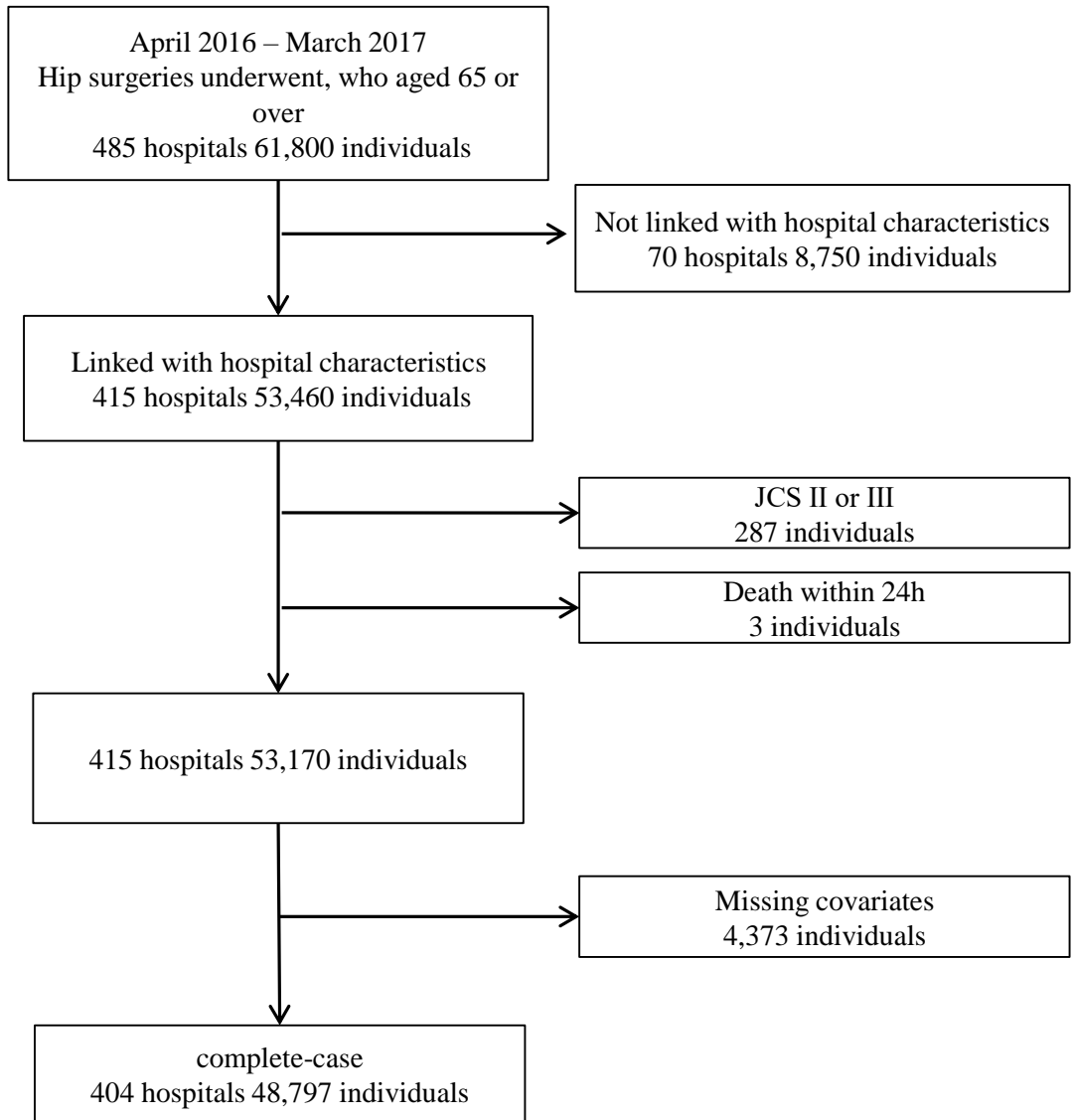

Supplement: S1 Fig — (PDF) [file pone.0249364.s001.pdf]
