# Supplementary material for: Dementia and patient outcomes after hip surgery in older patients: A retrospective observational study using nationwide administrative data in Japan
Source: PLoS One. 2021 Apr 22;16(4):e0249364. doi: 10.1371/journal.pone.0249364 (PMC8061936; doi:10.1371/journal.pone.0249364)
Supplement: S1 Table — (PDF) [file pone.0249364.s002.pdf]

S1 Table. Results of multivariate analyses for in-hospital death, in-hospital pneumonia, and in-hospital fracture and dementia (full model) (n=48,797)

	In-hospital death				In-hospital pneumonia				In-hospital fracture			
	OR	95% CI		P	OR	95% CI		P	OR	95% CI		P
**Fixed effect**												
With dementia (ref. without dementia)	1.12	0.95	1.33	0.181	0.95	0.51	1.80	0.885	1.08	0.92	1.25	0.344
Female (ref. male)	0.35	0.30	0.41	<0.001	0.29	0.16	0.52	<0.001	0.95	0.81	1.11	0.516
Age	1.04	1.03	1.06	<0.001	1.08	1.03	1.12	0.001	1.00	0.99	1.01	0.968
Body mass index	0.90	0.88	0.93	<0.001	0.87	0.79	0.95	0.003	0.96	0.94	0.98	<0.001
Charlson comordity index (ref.0)												
1	1.24	1.01	1.51	0.038	4.69	2.01	10.98	<0.001	1.34	1.15	1.57	<0.001
2	2.17	1.76	2.68	<0.001	3.51	1.30	9.47	0.013	1.59	1.31	1.93	<0.001
≧3	3.75	3.02	4.66	<0.001	3.98	1.34	11.82	0.013	2.03	1.62	2.53	<0.001
Place of residence before admission (ref. Home)												
Long-term care facility	1.01	0.83	1.22	0.942	0.85	0.40	1.79	0.669	0.66	0.54	0.81	<0.001
Other (hospital, clinic, etc.)	0.82	0.62	1.10	0.183	1.98	0.88	4.47	0.099	0.88	0.68	1.14	0.330
Psychotropic drug prescription (ref. non use)	1.41	1.19	1.67	<0.001	1.17	0.63	2.18	0.622	1.70	1.46	1.99	<0.001
Type of surgery (ref. Bipolar hip arthroplasty or Total hip arthroplasty)												
Osteosynthesis	1.75	1.48	2.08	<0.001	0.56	0.32	1.00	0.048	2.72	2.33	3.17	<0.001
Number of beds quartile (ref. 1st)												
2nd	0.98	0.76	1.26	0.882	2.31	0.91	5.83	0.077	1.33	0.79	2.23	0.285
3rd	0.97	0.76	1.26	0.844	0.88	0.29	2.65	0.814	1.80	1.07	3.05	0.028
4th	1.03	0.79	1.32	0.847	0.75	0.24	2.40	0.633	1.35	0.79	2.32	0.278
The number of patients per nurse and associate nurse	1.07	0.96	1.19	0.253	0.99	0.64	1.52	0.962	1.08	0.86	1.35	0.524
Percentage of nurses among all nursing staff	1.01	0.99	1.02	0.332	1.03	0.97	1.11	0.317	1.01	0.98	1.05	0.457

Addition to special care for dementia in the fee schedule (ref. none)												
Type 2 (trained nurses)	1.17	0.95	1.43	0.138	1.34	0.61	2.93	0.468	1.48	0.96	2.29	0.075
Type 1 (multidisciplinary dementia care team)	0.83	0.67	1.04	0.110	0.48	0.17	1.32	0.156	0.97	0.62	1.51	0.890
Types of establish organization of hospitals (ref. national)												
Public	1.36	0.97	1.91	0.077	2.20	0.50	9.62	0.296	1.23	0.64	2.36	0.539
Social	1.22	0.74	2.03	0.439	2.43	0.38	15.67	0.351	0.89	0.32	2.43	0.814
Private	1.51	1.04	2.20	0.032	2.91	0.60	14.15	0.186	2.86	1.36	6.05	0.006
Others	1.47	1.03	2.11	0.034	1.44	0.29	7.08	0.657	1.67	0.84	3.33	0.146
**Random effect**												
Intercept, hospital	0.15	0.08	0.30		1.49	0.62	3.58		2.46	2.02	3.00	

CI, confidence interval; OR, odds ratio
