# Supplementary material for: Dementia and patient outcomes after hip surgery in older patients: A retrospective observational study using nationwide administrative data in Japan
Source: PLoS One. 2021 Apr 22;16(4):e0249364. doi: 10.1371/journal.pone.0249364 (PMC8061936; doi:10.1371/journal.pone.0249364)
Supplement: S2 Table — (PDF) [file pone.0249364.s003.pdf]

S2 Table. Results of multivariate analyses for log (length of hospital stay) and dementia (full model) (n=48,049)

	Univariate				Multivariate			
	Coefficient	95% CI		P	Coefficient	95% CI		P
**Fixed effect**								
With dementia (ref. without dementia)	-0.047	-0.056	-0.039	<0.001	-0.007	-0.016	0.003	0.184
Female (ref. male)	-0.052	-0.062	-0.042	<0.001	-0.037	-0.047	-0.027	<0.001
Age	-0.001	-0.001	0.000	<0.01	0.003	0.003	0.004	<0.001
Body mass index	0.003	0.002	0.004	<0.001	0.001	0.000	0.002	0.110
Charlson comordity index (ref.0)								
1	0.019	0.010	0.029	<0.001	0.040	0.031	0.050	<0.001
2	0.066	0.053	0.078	<0.001	0.090	0.078	0.102	<0.001
≧3	0.148	0.132	0.164	<0.001	0.161	0.145	0.177	<0.001
Place of residence before admission (ref. Home)								
Long-term care facility	-0.227	-0.238	-0.216	<0.001	-0.228	-0.242	-0.214	<0.001
Other (hospital, clinic, etc.)	-0.114	-0.130	-0.098	<0.001	-0.112	-0.128	-0.096	<0.001
Place of residence after discharge (ref. Home)								
Long-term care facility	-0.199	-0.213	-0.186	<0.001	-0.103	-0.119	-0.087	<0.001
Other (hospital, clinic, etc.)	-0.099	-0.109	-0.089	<0.001	-0.113	-0.124	-0.103	<0.001
Psychotropic drug prescription (ref. non use)	0.077	0.069	0.086	<0.001	0.089	0.081	0.098	<0.001
Type of surgery (ref. Bipolar hip arthroplasty or Total hip arthroplasty)								
Osteosynthesis	-0.011	-0.019	-0.003	<0.01	0.011	0.002	0.020	<0.05
Number of beds quartile (ref. 1st)								
2nd	-0.089	-0.170	-0.008	<0.05	-0.041	-0.117	0.034	0.281
3rd	-0.193	-0.274	-0.111	<0.001	-0.146	-0.223	-0.069	<0.001
4th	-0.295	-0.376	-0.214	<0.001	-0.237	-0.315	-0.158	<0.001
The number of patients per nurse and associate nurse	0.082	0.045	0.119	<0.001	0.075	0.042	0.109	<0.001

Percentage of nurses among all nursing staff	-0.007	-0.012	-0.002	<0.01	0.000	-0.005	0.005	0.937
Addition to special care for dementia in the fee schedule (ref. none)								
Type 2 (trained nurses)	0.055	-0.020	0.129	0.149	0.045	-0.020	0.111	0.175
Type 1 (multidisciplinary dementia care team)	-0.063	-0.137	0.012	0.098	-0.020	-0.085	0.045	0.542
Types of establish organization of hospitals (ref. national)								
Public	0.041	-0.063	0.144	0.442	0.029	-0.067	0.126	0.550
Social	0.021	-0.141	0.184	0.798	-0.056	-0.201	0.089	0.451
Private	0.221	0.105	0.337	<0.001	0.129	0.016	0.242	<0.05
Others	0.147	0.037	0.257	<0.01	0.095	-0.008	0.197	0.070
**Random effect**								
Intercept, hospital					0.069	0.060	0.080	

The exact percentage change, %Δy = 100 * [exp (bjΔXj)−1], will give a more accurate prediction of the change in length of hospital stay.
CI, confidence interval.
